# Environmental burden of disease from unsafe and substandard housing, New Zealand, 2010–2017

**DOI:** 10.2471/BLT.20.263285

**Published:** 2021-02-01

**Authors:** Lynn Riggs, Michael Keall, Philippa Howden-Chapman, Michael G Baker

**Affiliations:** a Motu Economic and Public Policy Research, 97 Cuba Street, Wellington 6011, New Zealand.; b Department of Public Health, University of Otago, Dunedin, New Zealand.

## Abstract

**Objective:**

To assess the burden of disease related to unsafe and substandard housing conditions in New Zealand from 2010 to 2017.

**Methods:**

We focused on substandard housing conditions most relevant for New Zealand homes: crowding, cold, damp or mould, and injury hazards linked to falls. We estimated the population attributable fraction using existing estimates of the population exposed and exposure–response relationships of health disorders associated with each housing condition. We used government hospitalization data, no-fault accident insurance claims and mortality data to estimate the annual disease burden from the most severe cases, as well as the resulting costs to the public sector in New Zealand dollars (NZ$). Using value of a statistical life measures, we estimated the indirect cost of deaths.

**Findings:**

We estimated that illnesses attributable to household crowding accounted for 806 nights in hospital annually; cold homes for 1834 hospital nights; and dampness and mould for 36 649 hospital nights. Home injury hazards resulted in 115 555 annual accident claims. We estimated that direct public sector costs attributable to these housing conditions were approximately NZ$ 141 million (100 million United States dollars, US$) annually. We also estimated a total of 229 deaths annually attributable to adverse housing and the costs to society from these deaths at around NZ$ 1 billion (US$ 715 million).

**Conclusion:**

Of the conditions assessed in this study, damp and mouldy housing accounted for a substantial proportion of the burden of disease in New Zealand. Improving people’s living conditions could substantially reduce total hospitalization costs and potentially improve quality of life.

## Résumé

**Objectif:**

Évaluer l'impact qu'exercent les maladies imputables aux conditions de logement dangereuses et précaires en Nouvelle-Zélande entre 2010 et 2017.

**Méthodes:**

Nous nous sommes concentrés sur les conditions de logement précaires les plus fréquentes dans le pays: surpeuplement, froid, humidité ou moisissure, et risques de traumatismes causés par des chutes. Nous avons calculé la fraction attribuable dans la population en usant d'estimations existantes relatives à la population exposée et aux relations exposition-réponse des problèmes de santé associés à chaque type de logement insalubre. Nous avons également employé les informations gouvernementales concernant les hospitalisations, les demandes d'indemnisation sans égard à la faute et les données de mortalité afin de déterminer l'impact annuel des maladies les plus graves ainsi que les coûts engendrés pour le secteur public en dollars néo-zélandais (NZD) Enfin, nous avons établi le coût indirect des décès en utilisant les mesures de valeur d'une vie statistique.

**Résultats:**

Selon nos calculs, les maladies imputables aux logements surpeuplés ont entraîné 806 nuits d'hôpital par an; le froid régnant à l'intérieur des habitations a représenté l'équivalent de 1834 nuits d'hôpital; tandis que l'humidité et la moisissure ont débouché sur 36 649 nuits d'hôpital. Les risques de traumatismes survenant au domicile ont donné lieu à 115 555 demandes d'indemnisation chaque année. Nous estimons que les conditions de logement génèrent des coûts annuels directs d'environ 141 millions NZD (100 millions de dollars américains, USD) pour le secteur public. Nous pensons en outre qu'un total de 229 décès annuels sont liés à l'insalubrité des habitations, ce qui représente un coût de près de 1 milliard NZD (715 millions USD) pour la société.

**Conclusion:**

De toutes les situations examinées dans le cadre de cette étude, c'est la présence d'humidité et de moisissure qui a le plus d'impact sur la santé en Nouvelle-Zélande. Améliorer les conditions de logement de la population pourrait grandement contribuer à la diminution du coût total des hospitalisations et à une meilleure qualité de vie.

## Resumen

**Objetivo:**

Evaluar el impacto de la enfermedad relacionada con las condiciones de vivienda inseguras y precarias en Nueva Zelanda entre 2010 y2017.

**Métodos:**

Nos hemos centrado en las condiciones de precariedad de la vivienda más relevantes para los hogares neozelandeses: el hacinamiento, el frío, la humedad o el moho, y los riesgos de traumatismos relacionados con las caídas. Calculamos la fracción atribuible a la población utilizando las estimaciones existentes de la población expuesta y las relaciones exposición-respuesta de los problemas de salud asociados a cada tipo de infravivienda. También utilizamos información gubernamental sobre ingresos hospitalarios, reclamaciones de seguros de accidentes sin culpa y datos de mortalidad para estimar el impacto anual de las enfermedades más graves, así como los costes resultantes para el sector público en dólares neozelandeses (NZD). Por último, determinamos el coste indirecto de las muertes utilizando medidas de valor de una vida estadística.

**Resultados:**

Se calculó que las enfermedades causadas por el hacinamiento en el hogar suponían 806 noches de hospitalización al año; el frío en los hogares, 1.834 noches de hospitalización; y la humedad y el moho, 36.649 noches de hospitalización. Los riesgos por traumatismos en el hogar dieron lugar a 115.555 reclamaciones anuales por accidentes. Estimamos que las condiciones de la vivienda generan unos costes anuales directos para el sector público de aproximadamente 141 millones de NZD (100 millones de USD). Además, creemos que un total de 229 muertes al año están relacionadas con las malas condiciones de la vivienda, lo que cuesta a la sociedad casi 1.000 millones de dólares neozelandeses (715 millones de dólares estadounidenses).

**Conclusión:**

De todas las situaciones analizadas en este estudio, es la presencia de humedad y moho la que tiene mayor impacto en la salud en Nueva Zelanda. La mejora de las condiciones de la vivienda de la población podría contribuir en gran medida a reducir el coste total de la hospitalización y a mejorar la calidad de vida.

## ملخص

الغرض تقييم العبء المرضي المتعلق بظروف السكن غير الآمن ودون المستوى، في نيوزيلندا، خلال الفترة من 2010 إلى 2017.

الطريقة قمنا بالتركيز على ظروف السكن دون المستوى الأكثر شيوعاً في المنازل بنيوزيلندا: الازدحام، أو البرد، أو الرطوبة، أو العفن، ومخاطر الإصابة المرتبطة بالسقوط. قمنا بتقدير الشريحة السكانية المعنية باستخدام التقديرات الحالية للسكان المعرضين، والعلاقات المعتمدة على الاستجابة للتعرض، مع الاضطرابات الصحية المرتبطة بكل ظرف من الظروف السكانية. قمنا باستخدام البيانات الحكومية للعلاج بالمستشفيات، ومطالبات التأمين ضد الحوادث غير الخاطئة، وبيانات الوفيات، لتقدير عبء المرض السنوي من أشد الحالات سوءاً، بالإضافة إلى التكاليف الناتجة على القطاع العام بالدولار النيوزيلندي (NZ$). وباستخدام قيمة مقاييس الحياة الإحصائية، قمنا بتقدير التكلفة غير المباشرة للوفيات.

النتائج لقد قدرنا أن الأمراض التي تُعزى إلى الازدحام في المسكن قد نتج عنها 806 ليلة في المستشفى سنويًا؛ أدت المنازل الباردة إلى قضاء 1834 ليلة في المستشفى؛ وأدت والرطوبة والعفن إلى قضاء 36649 ليلة بالمستشفى. أدت مخاطر الإصابات المنزلية إلى 115555 مطالبة حوادث سنوية. وقد قدرنا أن التكاليف المباشرة للقطاع العام المنسوبة إلى ظروف السكن هذه كانت 141 مليون دولار نيوزيلندي تقريبًا (100 مليون دولار أمريكي) سنويًا. كذلك قدرنا إجمالي 229 حالة وفاة سنويًا تُعزى إلى الظروف المعاكسة للسكن، وكانت خسائر المجتمع من هذه الوفيات حوالي مليار دولار نيوزيلندي (715 مليون دولار أمريكي).

الاستنتاج من بين الظروف التي تم تقييمها في هذه الدراسة، شكلت المساكن الرطبة والمصابة بالعفن نسبة كبيرة من عبء المرض في نيوزيلندا. إن تحسين الظروف المعيشية للأشخاص يمكن أن يؤدي إلى تقليل التكاليف الإجمالية للعلاج بالمستشفى إلى حد كبير، ومن المحتمل أن يحسن جودة الحياة.

## 摘要

**目的:**

旨在评估 2010 年至 2017 年期间与新西兰不安全及不合格住房条件有关的疾病负担。

**方法:**

我们侧重于讨论与新西兰房屋最相关的不合格住房条件：拥挤、寒冷、潮湿或发霉，以及因跌倒导致的受伤风险。我们使用现有的人群暴露估计值以及与各种住房条件相关的疾病的暴露-反应关系来估计人群归因分值。我们使用政府报告的住院患者数据、无过错事故保险索赔和死亡率数据来估计最严重病例导致的年度疾病负担，以及由此产生的公共部门成本（以新西兰元计）。我们使用统计生命价值指标估计了死亡的间接成本。

**结果:**

我们估计每年有人因住房拥挤而导致的疾病住院 806 天；住房寒冷导致住院 1834 天；以及潮湿和发霉导致住院 36,649 天。家庭伤害危险导致每年 115,555 起事故索赔。我们估计每年因这些住房条件而产生的直接公共部门成本约为 1.41 亿新西兰元（1 亿美元）。我们还估计每年总共有 229 人因不良住房而导致死亡，这些死亡对社会造成的损失约为 10 亿新西兰元（7.15 亿美元）。

**结论:**

在这项研究所评估的条件中，潮湿和发霉的住房是导致新西兰绝大部分疾病负担形成的主要原因。改善人们的生活条件可以大幅降低住院总成本并有可能改善生活质量。

## Резюме

**Цель:**

Оценить бремя болезней, связанных с небезопасным и обветшалым жильем, в Новой Зеландии за период 2010–2017 гг.

**Методы:**

Авторы сосредоточились на условиях в обветшалых жилищах, которые наиболее актуальны для домов в Новой Зеландии: тесноте, холоде, сырости или плесени, а также опасности травм, связанных с падениями. Была рассчитана добавочная доля популяционного риска с использованием существующих оценок количества подверженного населения и взаимосвязей «подверженность-реагирование» для нарушений здоровья, связанных с каждым жилищным условием. Для этого использовались правительственные данные о госпитализации, страховые требования по случаям страхования без вины и данные о смертности, чтобы оценить годовое бремя болезней в наиболее тяжелых случаях, а также итоговые затраты для государственного сектора в новозеландских долларах. Используя статистические показатели продолжительности жизни, авторы оценили косвенные затраты в связи со смертью.

**Результаты:**

Было определено, что на долю болезней, связанных с проживанием в условиях скученности, приходилось 806 больничных суток ежегодно, а на долю болезней, связанных с проживанием в холодных помещениях, — 1834 больничных суток, госпитализация в связи с сыростью и плесенью в домах составила 36 649 больничных суток. Из-за травмоопасности жилищ было зафиксировано 115 555 жалоб ежегодно. По нашим оценкам, прямые затраты государственного сектора, связанные с такими жилищными условиями, составили приблизительно 141 миллион новозеландских долларов (100 миллионов долларов США) в год. Было также определено, что с неблагоприятными жилищными условиями в общей сложности ежегодно связаны 229 смертей и ущерб для общества от этих смертей составляет примерно 1 миллиард новозеландских долларов (715 миллионов долларов США).

**Вывод:**

Из условий, оцениваемых в рамках данного исследования, значительная часть бремени болезней в Новой Зеландии приходилась на сырость и плесень в жилищах. Улучшение условий жизни людей может существенно снизить общие расходы на госпитализацию и потенциально улучшить качество жизни.

## Introduction

In New Zealand, dampness, mould and cold are common in both owner-occupied and rental dwellings.^1^^–^^3^ In a government survey of 12 000 households in 2018–2019, 34% of New Zealanders reported that their homes were sometimes or always damp and 36% that their homes were mouldy.^4^ Temperature measurements in approximately 6700 New Zealand homes in 2020 found that one third of homes had an average daytime inside temperature below 18°C.^5^ In 2018, approximately half (388 310) of the 785 063 new injury claims due to falls in New Zealand happened in the home.^6^

Much of the literature examining the relationship between housing conditions and health disorders worldwide has focused on specific adverse housing conditions. A review of the evidence linking housing conditions to health provided examples of local public health activities to address these issues.^7^ However, we found few studies that assessed the total burden of disease from substandard housing conditions.^8^^–^^10^ One study made a cost estimate of the total burden as part of a cost–benefit analysis of making the housing stock in England healthy and safe.^10^ A study in the World Health Organization (WHO) European Region demonstrated that the environmental burden of disease approach is feasible for studying substandard housing conditions.^8^ None of these studies, however, detailed the burden from each health disorder or analysed which disorders were the biggest drivers of costs.

Here we estimate the burden of disease attributable to four substandard housing conditions found most frequently in New Zealand: household crowding, cold housing, damp or mouldy housing, and injury hazards linked to falls. We provide policy-makers with information to understand where resources might best be targeted and the potential benefits of policies undertaken to improve poor housing conditions.

## Methods

We used WHO methods of assessing the environmental burden of disease at the national level.^11^ We selected the household risk factors to analyse (crowding, cold, damp or mould, and hazards leading to falls) based on new recommendations in the 2018 WHO *Housing and health guidelines*.^12^ We did not consider housing conditions which had existing guidelines (toxic materials such as lead and asbestos; water, sanitation and hygiene; and indoor air pollution from solid fuels and noise) or where exposure in New Zealand is principally occupational (lead and asbestos).^13^^,^^14^ Although New Zealand has a guideline for dampness and mould, we included this condition because lack of insulation and low indoor temperatures affect the extent of dampness and mould.^12^ We focused on the aggregate burden of these household conditions because the solutions to these problems are not necessarily independent.

### Data sources

The first step was determining the proportion of the population exposed to the studied household risk factors in New Zealand homes. For household crowding, we used data from the national census on the proportion of the population reported to live in crowded conditions.^15^ For cold and dampness or mould, we obtained data from the New Zealand General Social Survey on the proportion of people reporting their home was colder than they would like and the proportion who had a problem with dampness or mould.^3^ For exposure to risk of falls, we used data from a randomized controlled trial about the proportion of homes in need of repair to prevent falls among a sample of houses typical of New Zealand housing.^16^ More details of the selection of risk factor exposures are presented in Table 1.

**Table 1:** Data sources used to estimate probability of exposure to adverse household conditions in New Zealand

Risk factor	Source	Study type and population	Measure	Proportion of people exposed
Household crowding	New Zealand Ministry of Health, 2014^15^	2013 Census of entire New Zealand population. Data on crowding covered 3 931 041 people	Proportion of population living in crowded conditions. A household was considered crowded if there was a one-bedroom deficit	10.1% (95% CI: 10.1–10.2%) all ages; 15.4% of children aged 0–4 years
Cold housing	Statistics New Zealand, 2015^3^	2014 General Social Survey of a representative survey of New Zealanders aged ≥ 15 years. 8795 individuals answered the personal questionnaire	Proportion of people surveyed who reported their home was always or often colder than they would like	21.2% (95% CI: 20.0–22.3%)
Damp or mouldy housing	Statistics New Zealand, 2015^3^	2014 General Social Survey of a representative survey of New Zealanders aged ≥ 15 years. 8795 individuals answered the personal questionnaire	Proportion of people surveyed who had a minor or major problem with dampness or mould in their home. Housing conditions were self-reported, with the presence of dampness and mould indicated by sight or smell (such as visible mould or dampness, mouldy or musty odour)	31.8% (95% CI: 29.7–33.8%)
Injury hazards leading to falls	Keall MD, et al., 2015^16^	Randomized controlled trial of 842 New Zealand households, 2009–2013. Households were randomly assigned to have either immediate home modifications done to prevent falls or to wait 3 years (436 in the treatment group and 406 in the control group)	Proportion of homes in need of repair to prevent falls among a sample of houses typical of New Zealand housing. 94% (382/406) of homes needed at least one modification	26% of home injuries caused by falls needing medical treatment were preventable by home modifications

CI: confidence interval.

The second step was obtaining data on health disorders associated with the studied housing conditions and the exposure–response measure (an odds ratio or relative risk) of each problem. We did not conduct a systematic review of the literature but used data from existing reviews or meta-analyses worldwide (Table 2). For crowding, indoor cold and injury hazards we relied principally on the systematic reviews which informed the WHO housing and health guidelines.^12^ For data on damp or mouldy housing, we used the WHO guidelines on dampness and mould.^27^ We selected health outcomes where the certainty of evidence in the systematic reviews was rated as medium or high. We included only falls in our assessment of injury hazards from home injuries as the WHO housing and health guidelines grade the quality of evidence for hazards other than falls as low or very low. More details of our criteria for selection of health disorders and exposure–response measures for the analysis are provided in Box 1.

**Table 2:** Data sources used to estimate the exposure–response measures for health outcomes attributable to household crowding, cold, and damp or mould

Housing condition by associated health disorder	ICD-10-AM diagnostic codes	Source^a^	Study type	Included data	Study population	Exposure–response measure (95% CI)
**Household crowding**						
Gastroenteritis	A0-A9, R11, K528, K529	Baker et al., 2013^17^	Meta-analysis of crowding studies based on keyword search	NA	Participants in 10 studies included in meta-analysis out of 81 reviewed	OR: 1.13 (1.01–1.26)
Pneumonia or lower respiratory tract infection^b^	B59, J09-J13, J15-J18, J20, J22, A481, A482	Grant et al., 2012^18^	Case–control study; crowding based on > 1 person per room	Hospitalizations or emergency department discharges with pneumonia diagnosis	Children aged 0–5 years	OR: 1.39 (0.78–2.48)
Upper respiratory tract infection	J00-J06, J32, J36, J37	Baker et al., 2013^17^	Meta-analysis of crowding studies based on keyword search	NA	Participants in 10 studies included in meta-analysis out of 90 reviewed	OR: 1.39 (0.69–2.79)
Meningococcal disease	A39	Norheim et al., 2014^19^	Cohort study; crowding based on two or more people per room excluding kitchen and bathroom for all and excluding living room in Sweden	Invasive meningococcal disease	Children aged 0–5 years in Norway, Sweden, Denmark and the Netherlands	OR: 1.05–1.07 (1.03–1.09)
Tuberculosis	A15-A19, J65, N740, N741	Baker et al., 2008^20^	Ecological study; crowding based on percentage of crowded houses in census area unit	Number of tuberculosis cases in census area unit	Participants in 1860 census area units in New Zealand	IRR: 1.05 (1.02–1.08)^c^
Influenza and influenza-like illness	J09-J11 (influenza)	Chandrasekhar et al., 2017^21^	Ecological study; crowding based on the percentage of crowded households (> 1 person per room) in the census tract	Laboratory-confirmed influenza-associated hospitalizations	33 515 hospitalizations in United States of America	OR: 1.17 (1.11–1.23)
Rheumatic fever	I00, I01, I02	Jaine et al., 2011^22^	Retrospective cohort study; proportion of crowded households in census area unit	Acute rheumatic fever cases	1249 cases in New Zealand	IRR: 1.07 (1.05–1.08)
**Cold housing**						
Chronic obstructive pulmonary disease symptoms^d^	J43, J44	Mu et al., 2017^23^	Cohort study; indoor temperature below 18.2 °C	Respiratory problems	82 adults (40–85 years old) with chronic obstructive pulmonary disease in China	Low humidity, OR: 1.032 (0.983–1.085); moderate humidity, OR: 1.024 (1.001–1.040); high humidity, OR: 1.087 (1.072–1.101)
Wheeze	R062 (wheeze), R061 (stridor)	Howden-Chapman et al., 2007^24^	Randomized trial; household received insulation (treatment group)	Self-reported wheezing in past 3 months	1350 households; 4 407 participants	OR: 1.75 (1.43−2.50)
Winter colds or influenza	J00 (common cold) J09-J11 (influenza)	Howden-Chapman et al., 2007^24^	Randomized trial; household received insulation (treatment group)	Self-reported winter colds or influenza	1350 households; 4 407 participants	OR: 1.85 (1.52–2.33)
**Damp or mouldy housing**						
Bronchitis	J20, J40, J41, J42	Fisk et al., 2010^25^	Meta-analysis; exposure based on reports of visible dampness and/or mould or mould odour as risk factors	NA	Participants in 13 studies	OR: 1.45 (1.32–1.59)
Respiratory infections^e^	J (ICD-10, chapter 10)	Fisk et al., 2010^25^	Meta-analysis; exposure based on reports of visible dampness and/or mould or mould odour as risk factors	NA	Participants in 19 studies	OR: 1.44 (1.31–1.59)
Asthma, current^f^	J45, J46	Fisk et al., 2007^26^	Meta-analysis; exposure based on reports of visible dampness and/or mould or mould odour as risk factors	NA	Participants in 10 studies	OR: 1.56 (1.30–1.86)
Asthma, ever-diagnosed	J45, J46	Fisk et al., 2007^26^	Meta-analysis; exposure based on reports of visible dampness and/or mould or mould odour as risk factors	NA	Participants in 8 studies	OR: 1.37 (1.23–1.53)
Asthma development	J45, J46	Fisk et al., 2007^26^	Meta-analysis; exposure based on reports of visible dampness and/or mould or mould odour as risk factors	NA	Participants in 4 studies	OR: 1.34 (0.86–2.10)
Wheeze	R062 (wheeze), R061 (stridor)	Fisk et al., 2007^26^	Meta-analysis; exposure based on reports of visible dampness and/or mould or mould odour as risk factors	NA	Participants in 22 studies	OR: 1.50 (1.38–1.64)
Cough	R05	Fisk et al., 2007^26^	Meta-analysis; exposure based on reports of visible dampness and/or mould or mould odour as risk factors	NA	Participants in 18 studies	OR: 1.67 (1.49–1.86)

CI: confidence interval; ICD-10-AM: *International statistical classification of diseases and related health outcomes, Tenth revision, Australian modification*; IRR: incident rate ratio; NA: not applicable; OR: odds ratio.

^a^ Original sources are cited in the World Health Organization (WHO) *Housing and health guidelines*, 2018,^12^ or *WHO guidelines for indoor air quality: dampness and mould,* 2009,^27^ except Fisk et al., 2010,^25^ which updated Fisk et al., 2007,^26^ and Baker et al., 2013,^17^ which used a meta-analysis to estimate exposure–response measures.

^b^ We removed bronchiolitis (code J21) and *Haemophilus influenzae* type B (code J14) from the pneumonia or lower respiratory tract infection category to avoid double counting.

^c^ In Braubach et al., the IRR used for tuberculosis and crowding was 1.5 (plausible range of 1.2 to 2.0) for European countries with medium and high tuberculosis incidence.^8^ We did not use this measure since it was not included in the WHO guidelines^12^ and New Zealand is a low tuberculosis incidence country. This rate is provided for comparison.

^d^ To be conservative, we use the low humidity odds ratio to calculate the population attributable fraction; however, we provide all the odds ratios for comparison.

^e^ Respiratory infections category excluded any diagnostic codes in this chapter that are specified as part of the other health disorders. For example, J45 and J46 are included under asthma and hence are not included in the broader respiratory infections category. Therefore, the categories are mutually exclusive and we can aggregate the health outcome measures without double counting.

^f^ Given that our outcome measures are hospitalizations, we expect that the current asthma odds ratio is the most relevant. The other odds ratios are provided for comparison.

Box 1 **Selection of health disorders and exposure–response measures due to substandard housing in New Zealand**

For determining which exposure–response measure to use when several studies were available, we selected studies based on three factors: (i) the quality of the research design; (ii) the exposure measure; and (iii) the similarity of the study population to New Zealand’s population. When looking at the quality of the research design, we ranked studies in the following order (starting with highest quality): meta-analysis; randomized controlled trial; well designed controlled trial without randomization; well designed cohort or case–control study; well designed ecological studies. In some instances where studies were of similar quality, we selected an exposure–response measure from a given study because the underlying population was more similar to New Zealand’s.

For dampness and mould, we included those health disorders with sufficient evidence of an association: asthma, upper respiratory tract infection, cough, wheeze, dyspnoea and respiratory infections.^27^ In addition, we included bronchitis because a subsequent meta-analysis^25^ indicated stronger evidence of an association between dampness and mould and bronchitis than was available for the World Health Organization (WHO) guidelines on dampness and mould.^26^ Moreover, since our outcome measure was principally hospitalizations, we excluded disorders that cannot be clearly linked to hospitalizations. For example, the quality of the evidence on the relationship between cold indoor temperatures and blood pressure is rated as high.^12^ However, since evidence on the relationship between cold and related hospitalizations was not available, we did not include blood pressure in the analysis.

For crowding, selection of data was complicated by studies using different crowding measures and very different populations. For tuberculosis risk due to household crowding, we did not use exposure–response measures based on research design quality and instead used data from an ecological study since it was for New Zealand. Moreover, the cohort and case–control studies that we would otherwise select had higher effect sizes. Hence, we may have underestimated tuberculosis outcomes, but this would not greatly change our results. We also deviated from the WHO guidelines on housing and health^12^ for upper respiratory tract infection and gastroenteritis due to difficulties in selecting appropriate studies. Instead, we used effect sizes from a meta-analysis for household crowding^17^ that was not included in the WHO guidelines.^12^ The effect sizes we used were generally lower than those found in the guidelines.^12^

The third step was obtaining data on outcome measures. For the health disorders associated with household crowding, cold and dampness, we obtained individual-level data from the health ministry on publicly funded hospitalizations and deaths.^28^ Specifically, we used administrative hospital admissions data from 2010 to 2017, including emergency department visits, for New Zealand residents where the primary diagnosis was associated with each of the studied health disorders. Diagnoses for each hospitalization are coded using the *International statistical classification of diseases and related health outcomes, Tenth revision, Australian modification* (ICD-10-AM).^29^ We considered hospital admissions within 7 days of another hospital discharge as part of the same case. For these cases, we used the primary diagnosis from the first hospitalization to assign a specific health disorder, and if the diagnosis did not match our list of diagnosis codes we used the second hospitalization diagnosis, and finally the third hospitalization diagnosis. By this method we assigned all events to only one health disorder so that outcomes were mutually exclusive, and no events were left unclassified. We also obtained health ministry data on deaths from each studied health disorder from 2010 to 2014, as data were only available up to 2014.^28^ These data included the underlying cause of death (coded using ICD-10-AM), age and ethnicity. 

For home injury hazards, we obtained administrative claims data for medically treated injuries from the government’s Accident Compensation Corporation which provides comprehensive coverage of the public-sector costs of accidents. Everyone in New Zealand is covered by this no-fault scheme if they are physically injured in an accident, with the scheme paying for medical treatment, lost wages, childcare, counselling, therapy and death benefits. We included claims for injuries between 2010 and 2017 where the scene of injury was listed as the home, the claimant was a resident, and the classification implied a fall.

### Data analysis

We estimated the population attributable fraction (*PAF*) for each health disorder using the following equation: 
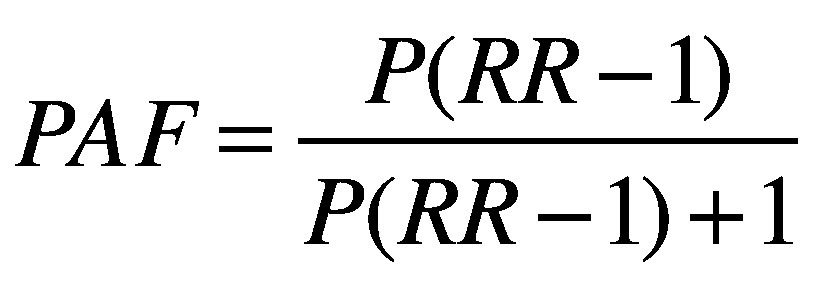
(1)where *P* is the proportion of the population exposed to the risk factor and *RR* is the exposure–response measure (odds ratio or relative risk) of the disease. This fraction represents the proportional reduction in adverse health outcomes that would occur if exposure to these risk factors was eliminated. Table 1 and Table 2 show the values of *P* and *RR* we used to calculate the population attributable fraction for indoor crowding, cold, dampness and mould. As children were over-represented in crowded households, we adjusted *P* for age when the associated health disorders were for specific ages. For falls, we used a population attributable fraction of 26% from other authors’ estimates.^16^

For health disorders linked to household crowding, cold or dampness or mould, we first determined the total length of stay and total cost of each hospitalization. Costs are in New Zealand dollars (NZ$), using 2017–2018 unit prices. The exchange rate during this time period was NZ$ 1 to 0.7148 United States $ (US$). We then aggregated these case-level estimates to estimate the number of hospitalizations, the number of unique patients, the total hospitalization cost and the total number of nights in hospital for each calendar year and then calculated the annual mean for each health disorder. For household crowding, some health disorders were specific to certain age groups. Hence, we used patient age to exclude hospitalizations outside the age range for these disorders. To estimate the burden of disease attributable to household crowding, cold and dampness or mould, we simply multiplied our population-level disease estimates by the population attributable fraction. 

Using individual death records, we used the ICD-10-AM codes for the underlying cause of death to classify deaths into the same categories as hospitalizations. We then aggregated the individual records to tabulate the total number of deaths for each health disorder in each calendar year and then calculated the annual mean to obtain the population-level estimates. Next, we multiplied the population-level estimates by the population attributable fraction to estimate the deaths attributable to household crowding, cold and damp or mould. 

For home injury hazards, we first used the claims data to estimate the population-level annual number of fatal and non-fatal fall injuries and the associated costs (such as medical treatment, lost wages, death benefits) from these injuries for each year in 2010–2017 by injury severity and then estimated the mean for this time period. The accident compensation scheme provided the data in six mutually exclusive injury severity categories for each claim. We used cases in the fatal injury category to estimate the number of deaths. Claim costs included all costs paid as at 1 October 2018 in NZ$. We then estimated the attributable burden by multiplying the population-level estimates by the population attributable fraction.

Finally, to estimate the societal costs of mortality attributable to all the studied household conditions, we used a willingness-to-pay based value of a statistical life.^30^ We estimated the value of a statistical life using a Value of Safety survey conducted in 1991 which asked New Zealanders about their willingness to pay for safety improvements that are expected to avoid one premature death.^31^ The 1991 value is indexed to average hourly earnings by the transport ministry to express it in 2017 NZ$.^31^

### Ethical considerations

Ethical approval for this research was granted by the University of Otago Ethics Committee (reference number HD18/094).

Access to anonymized data provided by Statistics New Zealand was in accordance with security and confidentiality provisions of the Statistics Act 1975. Only people authorized by the Statistics Act 1975 are allowed to see data about a particular person, household, business or organization and the results in this paper have been confidentialized to protect these groups from identification.

We carefully considered the privacy, security and confidentiality issues associated with using administrative and survey data in the Integrated Data Infrastructure. Further detail can be found in the Privacy impact assessment for the Integrated Data Infrastructure available from https://www.stats.govt.nz/about-us/.

## Results

### Population attributable fraction

The population attributable fraction for each health disorder linked to a housing condition is shown in Table 3. The highest population attributable fraction was for cough linked to dampness and mould (17.6%; uncertainty range: 12.7–22.5%) and the lowest was for tuberculosis from crowding (0.5%; uncertainty range: 0.2–0.8%). 

**Table 3:** Population attributable fractions for health disorders attributable to household crowding, cold, and damp or mould, New Zealand, 2010–2017

Housing condition by associated health disorder	Probability of exposure in the population, % (95% CI)^a^	Exposure–response measure (95% CI)^b^	Population attributable fraction, % (uncertainty range)
**Household crowding**			
Gastroenteritis	14.9 (14.9–14.9)	1.13 (1.01–1.26)	1.9 (0.1–3.7)
Meningococcal disease	12.5 (12.5–12.5)	1.05 (1.03–1.09)	0.6 (0.4–1.1)
Pneumonia or lower respiratory tract infection	14.9 (14.9–14.9)	1.39 (0.78–2.48)	5.5 (0.0–18.1)
Rheumatic fever (acute)	10.1 (10.1–10.2)	1.07 (1.05–1.08)	0.7 (0.5–0.8)
Rheumatic fever (chronic heart disease)	10.1 (10.1–10.2)	1.07 (1.05–1.08)	0.7 (0.5–0.8)
Tuberculosis	10.1 (10.1–10.2)	1.05 (1.02–1.08)	0.5 (0.2–0.8)
Upper respiratory tract infection	12.5 (12.5–12.5)	1.39 (0.69–2.79)	4.6 (0.0–18.3)
Influenza	10.1 (10.1–10.2)	1.17 (1.11–1.23)	1.7 (1.1–2.3)
**Cold housing**			
Chronic obstructive pulmonary disease symptoms	21.2 (20.0–22.3)	1.03 (0.98–1.09)	0.7 (0.0–1.9)
Wheeze	21.2 (20.0–22.3)	1.75 (1.43–2.50)	13.8 (7.9–25.1)
Winter colds or influenza	21.2 (20.0–22.3)	1.85 (1.52–2.34)	15.3 (9.3–22.8)
**Damp or mouldy housing **			
Bronchitis	31.8 (29.7–33.8)	1.45 (1.32–1.59)	12.5 (8.7–16.6)
Respiratory infections	31.8 (29.7–33.8)	1.44 (1.31–1.59)	12.3 (8.4–16.6)
Asthma, current	31.8 (29.7–33.8)	1.56 (1.30–1.86)	15.1 (8.2–22.5)
Wheeze	31.8 (29.7–33.8)	1.50 (1.38–1.64)	13.7 (10.1–17.8)
Cough	31.8 (29.7–33.8)	1.67 (1.49–1.86)	17.6 (12.7–22.5)

CI: confidence interval.

^a^ For some health disorders associated with household crowding, we use specific age groups. Hence, we adjusted the risk of exposure to account for the age group. See Table 1 for more details of the proportion of the population exposed.

^b^ See Table 2 for more details of the selection of exposure–response measures.

### Burden of hospitalization

The burden of disease estimates for household crowding, cold, and damp and mould are shown in Table 4. Overall, we estimated that 499 patients were hospitalized annually for illnesses attributable to household crowding (uncertainty range: 9–1722 patients). These patients accounted for 526 hospitalizations annually for a total of 806 nights costing almost NZ$ 1.4 million (US$ 1 million). The most expensive conditions attributable to household crowding were respiratory infections (pneumonia or lower respiratory tract infection and upper respiratory tract infection). While almost twice as many patients were hospitalized with upper respiratory tract infection than with pneumonia or lower respiratory tract infection (274 versus 156 patients), upper respiratory tract infection hospitalization costs were slightly lower (NZ$ 0.5 million versus NZ$ 0.6 million; US$ 0.39 million versus US$ 0.43 million).

**Table 4:** Health outcomes and related costs attributable to household crowding, cold, and damp or mould, New Zealand, 2010–2017

Housing condition by primary diagnosis	Age group, years	Population attributable fraction, %	Estimated no. of patients (uncertainty range)	Estimated no. of attributable hospitalizations (uncertainty range)	Estimated hospitalization costs, NZ$ (uncertainty range)	Estimated length of stay, no. of nights (uncertainty range)
**Household crowding**						
Gastroenteritis	0–5	1.9	63 (5–124)	66 (5–129)	148 041 (11 591–290 561)	90 (7–177)
Meningococcal disease	0–16	0.6	0.2 (0.1–0.4)	0.2 (0.1–0.4)	2 899 (1 743–5 192)	2 (1–3)
Pneumonia or lower respiratory tract infection	0–5	5.5	156 (0–515)	169 (0–555)	595 174 (0–1 958 065)	372 (0–1 223)
Rheumatic fever (acute)	≥ 0	0.7	1 (1–1)	1 (1–1)	15 223 (10 895–17 551)	17 (12–20)
Rheumatic fever (chronic heart disease)	≥ 0	0.7	3 (2–4)	4 (3–4)	95 465 (68 327–110 064)	38 (27–44)
Tuberculosis	≥ 15	0.5	1 (0–1)	1 (0–1)	11 066 (4 440–17 825)	13 (5–21)
Upper respiratory tract infection	0–18	4.6	274 (0–1076)	286 (0–1123)	542 207 (0–2 132 716)	274 (0–1 076)
Influenza	≥ 0	1.7	28 (18–37)	28 (18–38)	136 847 (89 079–185 830)	108 (70–147)
Total	NA	NA	499 (9–1 722)	526 (9–1 814)	1 410 075 (96 996–4 531 974)	806 (53–2 565)
**Cold housing**						
Chronic obstructive pulmonary disease symptoms	NA	0.7	48 (0–132)	69 (0–190)	546 058 (0–1 500 901)	594 (0–1 632)
Wheeze	NA	13.8	260 (149–473)	295 (169–536)	505 037 (289 176–918 127)	243 (139–441)
Winter colds or influenza	NA	15.3	260 (158–387)	261 (159–389)	1 262 283 (770 787–1 882 763)	997 (609–1488)
Total	NA	NA	568 (307–992)	625 (328–1 115)	2 313 378 (1 059 964–4 301 792)	1 834 (748–3 561)
**Damp or mouldy housing**						
Asthma, current	NA	15.1	900 (487–1 340)	1095 (593–1 632)	2 726 778 (1 475 772–4 062 618)	1 967 (1 064–2 930)
Bronchitis	NA	12.5	141 (98–188)	143 (99–190)	653 636 (453 165–868 116)	653 (453–867)
Cough	NA	17.6	93 (68–120)	96 (69–123)	268 726 (194 373–344 577)	137 (99–176)
Other respiratory infection	NA	12.3	174 (120–236)	177 (122–240)	1 746 705 (1 199 725–2 365 983)	1 582 (1 087–2 143)
Bronchiectasis	NA	12.3	97 (67–131)	138 (95–187)	836 869 (574 804–1 133 573)	957 (658–1 297)
Bronchiolitis	NA	12.3	424 (292–575)	541 (372–733)	2 149 708 (1 476 528–2 911 866)	1 166 (801–1579)
Pneumonia or lower respiratory tract infection	NA	12.3	2 316 (1 591–3 138)	2 486 (1 707–3 367)	23 736 929 (16 303 723–32 152 633)	28 253 (19 406–38 270)
Upper respiratory tract infection	NA	12.3	1 261 (866–1 709)	1 308 (898–1 771)	3 216 949 (2 209 563–4 357 488)	1 692 (1 162–2 292)
Wheeze	NA	13.7	259 (191–335)	293 (217–380)	502 503 (371 471–651 439)	242 (179–313)
Total	NA	NA	5 666 (3 779–7 771)	6 276 (4 171–8 622)	35 838 804 (24 259 125–48 848 294)	36 649 (24 908–49 868)

NA: not applicable; NZ$: New Zealand dollars.

Notes: Costs in NZ$ were estimated using 2017–2018 prices. The exchange rate during this time period was NZ$ 1 to United States $ 0.7148.

We estimated that 625 hospitalizations annually were attributable to living in cold homes (Table 4), accounting for 1834 nights in hospital and costing more than NZ$ 2.3 million (US$ 1.6 million; uncertainty range: NZ$ 1.1 million to 4.3 million). In this group, hospitalizations for cold or influenza had the highest cost at more than NZ$ 1.3 million (US$ 0.9 million). However, the same number of patients (260 patients) were hospitalized for wheeze as for colds or influenza but with 40% of the costs (NZ$ 0.5 million; US$ 0.4 million) and one quarter of hospital nights (243 versus 997 nights). Chronic obstructive pulmonary disease, on the other hand, accounted for 48 patients with 69 hospitalizations, but with costs similar to those for wheeze (NZ$ 0.5 million; US$ 0.4 million). This difference may be due to longer hospital stays for chronic obstructive pulmonary disease (8.6 nights per hospitalization) compared with cold or influenza (3.8 nights per hospitalization) or wheeze (0.8 nights per hospitalization).

We also found that more hospitalizations between 2010 and 2017 were attributable to dampness and mould than to cold or crowding (Table 4). In total, we estimated that annually 6276 hospitalizations of 5666 patients were attributable to dampness and mould accounting for 36 649 hospital nights costing almost NZ$ 36.0 million (US$ 25.7 million). Moreover, pneumonia or lower respiratory tract infection was the largest contributor to these totals, with 2486 hospitalizations annually for 28 253 nights costing approximately NZ$ 23.7 million (US$ 16.9 million). The second most costly condition was upper respiratory tract infections. Annual upper respiratory tract infection hospitalizations were about half of those for pneumonia or lower respiratory tract infection (1308 versus 2486 hospitalizations) but costs were about one tenth (NZ$ 3.2 million; US$ 2.3 million). This cost difference may be due to fewer nights in hospital; the average patient with pneumonia or lower respiratory tract infection spent 11.4 nights in hospital, whereas the average patient with upper respiratory tract infection spent just over one night.

The estimates of the burden of home injuries are shown in Table 5. In total, we estimated that claims from home injury hazards cost almost NZ$ 102.3 million (US$ 73.1 million) for 115 555 claims annually. Most claims (108 264 claims) only paid medical fees and cost almost NZ$ 30.8 million (US$ 22.0 million). Entitlement claims, which included medical fees plus other compensation (such as for loss of earnings, attendant care, home modifications) cost the most (NZ$ 35.8 million, US$ 25.6 million, annually for 5526 claims). Still, hospitalization claims cost almost NZ$ 30.0 million (US$ 21.4 million) annually even though they were about one quarter the number of entitlement claims.

**Table 5:** Home injury claims for falls and related costs (total and attributable), New Zealand, 2010–2017

Claim type	Annual mean for all home injury claims^a^			Estimated annual burden of disease attributable to home injury hazards	
	No. of claims	Cost, NZ$		No. of claims	Cost, NZ$
Fatal	280	5 225 388		68	1 277 085
Serious injury	49	17 218 369		12	4 208 169
Hospitalization	6 345	122 677 291		1 551	29 982 330
Entitlement claims	22 611	146 400 741		5 526	35 780 341
Medical fee only	442 977	125 979 878		108 264	30 789 482
Other	552	1 074 288		135	262 556
**Total**	**472 813**	**418 575 955**		**115 555**	**102 299 963**

NZ$: New Zealand dollars.

^a^ Claims for slips, trips or falls.

Notes: Costs in NZ$ were estimated using 2017–2018 prices. The exchange rate during this time period was NZ$ 1 to United States $ 0.7148.

### Burden of deaths

Overall, we estimated approximately 68 deaths were attributable to falls (Table 5), one death to household crowding, 16 deaths to cold and 145 deaths to dampness or mould (Table 6), for a total of 229 deaths annually. Using the value of a statistical life of NZ$ 4.2 million (June 2017 NZ$),^31^ we estimated the total cost of these deaths due to unsafe and substandard housing conditions to be NZ$ 938.9 million (US$ 671.1 million) annually.

**Table 6:** Annual mortality attributable to household crowding, cold, and damp or mould, New Zealand, 2010–2014

Housing condition by primary diagnosis	Average annual no. of deaths	Population attributable fraction, %	Estimated annual no. of attributable deaths (uncertainty range)
**Household crowding**			
Influenza	35	1.7	0.6 (0.4–0.8)
Rheumatic fever	122	0.7	0.9 (0.6–1.0)
Total	157	0.9	1.4 (1.0–1.8)
**Cold housing **			
Chronic obstructive pulmonary disease	1555	0.7	10.5 (0.0–28.8)
Cold or influenza	36	15.3	5.4 (3.3–8.1)
Total	1590	1.0	15.9 (3.3–36.9)
**Damp or mouldy housing**			
Asthma, current	73	15.1	11.0 (6.0–16.4)
Bronchitis	13	12.5	1.6 (1.1–2.1)
Other respiratory infection	296	12.3	36.3 (24.9–49.1)
Bronchiectasis	101	12.3	12.4 (8.5–16.8)
Pneumonia or lower respiratory tract infection	675	12.3	82.8 (56.9–112.1)
Upper respiratory tract infection	6	12.3	0.7 (0.5–0.9)
Total	1162	12.5	144.7 (97.8–197.5)

## Discussion

We estimated that approximately 8300 hospitalizations (0.4% of nearly 2 million annual average hospitalizations over the same time period, 2010–2017) and 229 deaths each year were attributable to substandard or unsafe housing conditions in New Zealand. Dampness and mould imposed the largest disease burden in terms of hospitalizations and deaths compared with the other housing conditions we analysed. Comparing costs across all housing conditions is difficult, since we have more complete information for injuries due to the comprehensive nature of the claims data. Even so, claims costs for hospitalized injury cases (almost NZ$ 30 million) – which include all costs paid in relation to the claim and not just the cost of hospitalization – were still exceeded by the estimated hospitalization costs attributable to damp and mouldy housing (around NZ$ 36 million). 

Our total annual cost estimate includes only direct costs to the public sector through hospitalization costs and injury claim payments. We estimated the mortality costs separately but did not include other costs for morbidity such as productivity losses from missed work, other than the earnings paid through injury claims. However, even this value underestimates total lost wages since only 80% of earnings are covered by the accident compensation scheme.^32^ From other studies, we also know that the total costs to society are likely to be much higher than the costs estimated here. For example, research valuing disability-adjusted life years using the value of a statistical life estimates the total cost of preventable home falls at around NZ$ 22 billion (in 2012 NZ$).^33^

Comorbidities may exacerbate the impact of these housing conditions on health, and many homes have overlapping issues, meaning that the health disorders resulting from these housing conditions may not be simply additive. For example, many cold homes are also damp or mouldy. We reported outcomes attributable to cold separately from damp and mould, even though the conditions are related,^34^ because the exposure–response rates for the health disorders are linked to one condition. One report suggested that many New Zealand homes would experience far fewer periods of high relative humidity if they were heated to a minimum of 18 °C throughout.^34^ However, we were unable to find any research that measured the interaction of these effects or the effect of comorbidities on these disorders. Hence, we did not account for the interaction of multiple housing conditions or comorbidities in our estimation of health disorders. 

Other studies have attempted to assess the overall burden and societal costs from unsafe and substandard housing conditions.^9^^,^^10^ An estimate of the costs of poor housing conditions in England showed that the most hazardous conditions in English homes cost the National Health Service (NHS) in excess of 600 million British pounds (£) per year, with the cost to society in excess of £1.5 billion per annum.^10^ Updated cost estimates, published in 2015, showed a £1.4 billion per annum cost for the NHS, putting the cost on par with those from physical inactivity, smoking or alcohol.^9^ Our estimates are of a similar magnitude given differences in the size of the two populations and currency exchange rates.

Our estimates of the disease burden are conservative since we do not have information about the number of nonhospitalized people for many of the housing-related health disorders we included (injuries are the exception), and hence, the costs do not include general practitioner visits or pharmaceutical costs. Moreover, the costs included in our analysis are primarily costs to the public sector from hospitalizations and generally do not include other social costs, except for mortality. For injuries, we included costs paid related to the claim (including wages paid for time off work), but these costs do not include other costs to society, only direct outlays.

Basing our estimates on self-reports of 21.2% of the population exposed to cold and 31.8% exposed to damp or mould may underestimate the true exposure as householders in New Zealand typically report better conditions than assessors’ reports for the same homes. For example, in one survey of 560 houses, assessors reported visible mould in 49% of houses.^1^ Other surveys recorded night-time temperatures below 18 °C in 84% of bedrooms (83 homes),^35^ and daytime temperatures below 18 °C in 33% of homes (6700 homes).^5^ Moreover, in homes with recorded daytime temperatures below 16 °C (15.1% of homes surveyed in winter), only 36% of householders thought their homes were always or often cold in winter and 45% were able to see their breath inside.^5^ Nevertheless, subjective measures of poor housing have been consistently linked with a significantly increased risk of health effects.^36^^,^^37^ We therefore believe that using self-reported or other subjective measures of dampness and mould for our exposure measure is supported by current research.

Our study is also likely to underestimate the true burden of poor housing since our data were limited to hospitalizations and deaths for most housing conditions (except fall injuries). For health disorders associated with the other housing conditions, most patients will never be hospitalized and some may never seek medical care, leading to undercounting of cases. For the direct costs to the public health-care sector, however, hospitalizations are likely to capture a substantial portion of the costs. In one study of housing-related health disorders for children, the majority of health care costs averted were due to reductions in hospitalizations, despite far greater numbers of general practitioner visits and prescriptions.^38^

Our analysis has highlighted gaps in the evidence. Further research is needed to better understand the interaction effects of different housing conditions and the resultant health effects. Moreover, more work is required to fully estimate the total burden, including the total cost to society. Future work would also benefit from a focus on vulnerable populations (such as home renters, low-income households) and the potential impact on inequality. Still, our estimates could be used to target policies at specific populations. For example, given the high cost of pneumonia or lower respiratory tract infection for household dampness and crowding, working to improve living conditions for these patients could substantially reduce total hospitalization costs and potentially improve their quality of life.
